# In the Swim of Cannabis: Developmental Toxicity and Metabolomic Pathway Alterations of Zebrafish Larvae Exposed to THC for the Assessment of Its Potential Environmental and Human Health Impact

**DOI:** 10.3390/molecules27175506

**Published:** 2022-08-27

**Authors:** Theodoros Chatzimitakos, Ieremias Chousidis, Dimitrios Leonardos, Constantine Stalikas, Ioannis Leonardos

**Affiliations:** 1 Laboratory of Analytical Chemistry, Department of Chemistry, University of Ioannina, 45110 Ioannina, Greece; 2 Laboratory of Zoology, Biological Applications and Technology Department, University of Ioannina, 45110 Ioannina, Greece; 3 School of Medicine, University of Ioannina, 45110 Ioannina, Greece

**Keywords:** tetrahydrocannabinol, zebrafish, toxicity, metabolomics, behavior, developmental toxicity

## Abstract

As the pharmacological properties and therapeutic applications of *Cannabis sativa L.* pace with the upsurge of interest of the scientific community in harnessing its constituent phytocannabinoids, illicit use may raise serious health issues. Tetrahydrocannabinol (THC) is one of the most well-known phytoactive constituents of cannabis and continues to garner scientific and public attention not only because of its pharmacological value but also because over-the-counter products of THC and prescription medications are becoming increasingly available from pharmacies, dispensaries, Internet, local retail stores, or by illicit means. Hence, a multidimensional approach was employed to examine the impact of THC on zebrafish larvae. The acute toxicity, expressed as LC_50_, was 1.54 mg/L. Adverse effects were observed on the phenotype, such as tail bending, pericardial edema, etc., even at concentrations lower than LC_50_, and fundamental functions of larvae (e.g., heart rate and cardiac contractility, and rhythm) were significantly affected. Behavioral changes were noticed, which were reflected in locomotor activity and sensitivity to light/dark changes. Finally, an untargeted metabolomic study was carried out to shed light on the metabolic alterations that occurred, providing substantiating evidence of the observed phenotype alterations. Overall, the potentially detrimental effects of THC on a vertebrate model are depicted.

## 1. Introduction

The relationship of humans with *Cannabis sativa* L. dates back thousands of years [1]. The improvident recreational use, which led to the prohibition of every cannabinoid use for many years, has alienated the scientific community to test possible therapeutic and adverse effects that could result from its use. Today, the current knowledge supports that nearly 500 compounds can be identified and isolated from the plant [2]. The main constituents of the plant are tetrahydrocannabinol (THC) and cannabidiol (CBD), at concentrations ranging between 0.1 and 25% and 0.1 and 2.89% (expressed as *w*/*w* of the dry weight of the plant), respectively [3]. Apart from their use for recreational purposes, e.g., euphoria [4], cannabinoids are currently being used extensively for the treatment of a series of syndromes, including epilepsy [5], Dravet syndrome [6], anxiety [7], schizophrenia [8], chronic pain [9], even for cancer [10]. The extensive use for such syndromes is justified by the proven beneficial and therapeutic effects. Recently, cannabinoids have been proposed for anti-inflammatory treatment in SARS-CoV-2, as it has been proven to be effective in suppressing immune and inflammatory functions [11,12,13,14].

The debate around the legalization and/or decriminalization of cannabis has been a bone of contention for many years. As more countries legalize recreational and medicinal cannabis, there is an urgent need to gather as much information about the main phytocannabinoids, as never before. A lot of studies have assessed the deleterious effects of the illicit use of cannabinoids in vertebrates, in which the endocannabinoid system is highly conserved [15]. The results from these studies demonstrated developmental, teratogenic, and behavioral abnormalities and most of these effects were more pronounced after exposure to the early-life stages [16,17,18,19]. Zebrafish is an important model organism that is widely used in pharmacogenetics, neuropharmacology, and environmental studies [20]. Recently, it has been used as a model organism in the study of cannabinoids [21] because it expresses both CB1 and CB2 receptors and all the other major endocannabinoid-related genes, even at very early-life stages [16]. The endocannabinoid gene function of zebrafish has been associated with addiction, anxiety, development, energy homeostasis and food intake, immune system function, learning, and memory [21]. Since zebrafish embryos reside in an aqueous environment, almost any water-soluble chemical can easily be administered to developing embryos to monitor their effects on development. At the same time, zebrafish can readily be used in real-time in vivo studies to address potential hazards to human health and can improve the limited understanding of the specific effects of exposures.

As the investigation of quasi-legal commodities is challenging, a number of peer-reviewed studies documenting the impact of cannabis has been released. Taking into account that there are reports for mild or severe effects of cannabinoids on organisms, such as zebrafish (*Danio rerio*) [16,17,18,19], there is a need for a preliminary multidimensional approach to these effects. Of the individual components, THC is the most comprehensively documented, as the extracted THC has become a larger share of the cannabis market. THC is lipophilic and is absorbed efficiently across body membranes. It distributes rapidly to vascular organs and is accumulated in adipose tissue. Importantly, it can cross the placenta and be conveyed to breast milk.

The aim of this study is to explore the effect of THC on zebrafish embryos in acute exposure regime, during critical developmental stages, by studying the systemic toxicity, behavioral implications, and metabolic pathways. An in-depth comprehension of the above will assist in quantifying the impacts and strengthening the understanding of cannabis profound implications on human health. The study can identify the knowledge gaps and increase our current limited knowledge of the consequences of exposure to a cannabis-related chemical like THC.

## 2. Materials and Methods

### 2.1. Chemicals

A THC (Δ9-Tetrahydrocannabinol) solution at a concentration of 10 mg/mL in ethanol was purchased from Lipomed AG (Arlesheim, Switzerland). All solvents used were of analytical grade. Deuterated water (D_2_O) and 3-(trimethylsilyl)-1-propionic-2, 2, 3, 3-*d*_4_ acid sodium salt (TSP) were obtained from Deutero (Deutero GmbH, Kastellaun, Germany).

### 2.2. Zebrafish Housing and Husbandry

Adult zebrafish of the wild-type strain (AB) were maintained in a colony room, in a recirculated system, at 28 ± 1 °C, pH 6.5–7.5, water conductivity of 500 ± 50 μS cm^−1^ with a 14-h light/10-h dark photoperiod (lights on at 8:00 a.m.). Feeding of the fish was performed twice a day with zebrafish feed, following common practices. Sexually mature zebrafish (at least three months old) were used for spawning. Embryos were collected and pooled into standard zebrafish E3 culture medium (5 mmol/L NaCl, 0.33 mmol/L CaCl_2_, 0.33 mmol/L MgSO_4_·7H_2_O, and 0.17 mmol/L KCl). 

### 2.3. Zebrafish Toxicity Testing

The collection of zebrafish eggs was performed at the beginning of the 14-h light phase following the mating procedure that took place overnight. After inspecting them, the unfertilized eggs and those that showed developmental disorders were removed. The dechorionation process of the eggs followed at 24 h post-fertilization (hpf). Details about the toxicity testing are given in the Supplementary Material.

### 2.4. Lethal Concentration (LC_50_) Determination

Toxicity assays (LC_50_ values) and confidence limits (LC_25_ and LC_75_) were calculated based on cumulative mortality at the end of the experiment. The LC_50_ values were assessed using Regression Probit analysis (the chi-square test, Pearson goodness of fit test, and 95% confidence interval).

### 2.5. Heart Rate

The effect of THC on the heart rate as a function of THC concentration and hpf were analyzed as a means to evaluate the effect on developmental ontogeny. Twenty embryos per concentration were separately analyzed under a microscope and a stereoscope (Olympus SZX7 Stereo, Olympus KL300 LED light), and high-quality video recordings were obtained for 1 min using Basler MED ace camera and Basler Microscopy software (Basler MED ace 2.3 MP 164 color, Basler Microscopy Software Version 2.1; Basler, Ahrensburg, Germany). The video recording frame rate was set to 25 fps. To eliminate any effect of temperature on the zebrafish heartbeat, the dissecting microscope was installed in a temperature-controlled room (28 °C) and video recordings were performed using transmitted cold lighting (LED). The acclimatization period was set at 20 min before each recording. The heart rate of embryos, under the effect of various concentrations of THC, was investigated on the second, third, and fourth day (48, 72, and 96 hpf, respectively) after fertilization. The effect of THC concentration on heart rate was assessed with the use of general linear model (GLM) analysis, using the concentrations of THC as an independent variable and the time (hpf) as a covariate. Statistical analyses were performed using the SPSS statistical software v26 (IBM Corp., Armonk, NY, USA).

For toxicity and heart rate no positive control was used for two reasons: first, zebrafish embryos were exposed to THC after the chorion had been removed in order to avoid the risk of generating false negative results in toxicity studies due to limited permeability of the chorion to some compounds [22]. Secondly, the toxicity of THC (LC_50_), carried out by exposing a large number of zebrafish embryos to various concentrations of THC, showed (*vide infra*) that mortality was dose-dependent and practically lower than 30%, at a concentration < 1.25 mg/L [23].

### 2.6. Behavior Screening

It is common practice to evaluate the alterations in the behavioral activity of zebrafish when testing the bioactivity of drugs. The effect of THC was investigated on the motor function and reflexes of the zebrafish embryos through behavioral screening. To this end, motor function was analyzed in relation to THC concentration (non-exposed and THC-exposed larvae) by performing various behavioral trials.

#### 2.6.1. Touching Motor Response (TMR)

The touching motor response (TMR) was evaluated by performing a light touch stimulus test applied to the rostral head area by a glass capillary injection needle to determine the responsivity of larvae to this stimulus. Larvae lacking any response to a touch or phenotypically being unable to swim were excluded from further behavioral assessment. 

#### 2.6.2. Locomotor Activity

To explore any developmental effect after exposure to THC, Basal Locomotor Activity (BLA) and Visual Motor Response (VMR) were evaluated at 6 days post-fertilization (dpf). To achieve this aim, 100 larvae per concentration and 120 non-exposed ones, as vehicle control, were transferred to an isolated behavioral screening room to assess the larval swimming activity (i.e., distance moved, acceleration) and the ability to adapt to changing environmental conditions (dark/light cycling). By testing this adaptation, the quantification of the behavior in two distinct environments is possible. The larvae were placed in 24-well plates with 1.5 mL of E3 embryo buffer in each well. Then, the plates were transferred to a behavioral testing chamber with a temperature control unit (DanioVision, Noldus Inc., Wageningen, The Netherlands) and kept in dark, at 28 °C, for 1 h, for acclimatization. The position of each larva was recorded by an IR digital video camera Basler acA1300-60gm (Basler Inc., Exton, PA, USA). Any background noise was removed by setting a minimum distance input with a filter of 10% of the larva body, equivalent to 0.4 mm. The locomotor activity was tracked in a 30-min trial period as follows: 10 min dark, 10 min light, and 10 min dark. This tracking circle was followed to analyze the ability of the embryos to adapt to changing environmental stimuli (i.e., alternating phases of light and dark).

The video recordings were held between 10 am and 12 pm to stabilize zebrafish basal activities related to circadian rhythms. The distance moved and the velocity were recorded with EthoVision XT tracking software (ver. 14 Noldus, Wageningen, The Netherlands). The difference in activity between treatment groups and vehicle controls regarding the distance moved (cm), the velocity (cm/s), and the impact of the dark/light cycling on those two parameters were analyzed with the GLM. Any differences were considered significant at a probability level of 0.1%, as a minimum criterion of significance.

#### 2.6.3. Vibrational Startle Response (VSR)

The vibrational startle response (VSR) was studied by investigating the tap-elicited startle reflex performed by the Tapping Device system (DanioVision Tapping Device DVOC-004x/T) installed in a Noldus DanioVision Observation Chamber (Noldus, Wageningen, The Netherlands). 

### 2.7. Metabolomic Study and Data Processing

#### 2.7.1. Metabolite Extraction

The procedure followed is similar to that of our previous study [17]. In brief, larvae (144 hpf) were transferred to falcon tubes and washed with water. After euthanizing the larvae by snap freezing, the samples were lyophilized. Metabolites were extracted following the Bligh and Dyer extraction method. Briefly, chloroform (1 mL) and methanol (2 mL) were added to the dry samples and after mixing chloroform (1 mL) and water (1 mL) were added. After ultrasonication and vortex mixing, the samples were centrifuged (3000 rpm for 5 min). The upper phase was collected, divided into two equal portions, and evaporated to dryness using a gentle nitrogen stream. In the first sub-sample (intended for NMR spectra) 600 μL of D_2_O (containing TSP) was added and transferred into an NMR tube. To the second sub-sample (for LC-HRMS analysis) 100 μL acetonitrile was added.

#### 2.7.2. Metabolome Study and Data Processing

After correcting the ^1^H-NMR spectra shifts, based on the chemical shift of TSP, the chemical shifts were recorded and entered in the Human Metabolome Database (1D NMR search engine), as well as in the NMR search engine of Madison Metabolomics Consortium Database. The metabolites generated from both databases were recorded and the common metabolites were further identified within the MS spectra (by searching for the accurate masses of the compounds, using four decimals, and matching the isotopic distributions and fragmentation patterns with the database data). Finally, metabolites were introduced in the Metaboanalyst database and the metabolic pathways were recorded, using the “*Danio rerio* (zebrafish)” pathway library. Details about the instrumentation regarding the metabolomic study are given in the Supplementary Material.

### 2.8. Statistical Analyses 

Graphs and statistical analyses were processed by SPSS statistical software v26 (IBM Corp.). The lethal concentration (LC_50_) values were assessed using Regression Probit analysis (chi-square test, Pearson goodness of fit test, 95% confidence interval). The effect of THC concentration on heart rate (dependent variable) was assessed with the aid of GLM analysis using the concentration as the independent variable and hpf as a covariate. In order to explore the locomotor activity of larvae, the distance moved and the velocity were recorded with EthoVision XT tracking software (ver. 14 Noldus, Wageningen, The Netherlands). The differences in locomotor activity regarding dark/light cycling were analyzed with the GLM using concentration as a dependent variable and time as a covariate. The vibrational startle response (VSR) test was performed with the aid of the EthoVision XT tracking software. Differences were considered significant at *p* < 0.001 and marginally significant at *p* < 0.05.

### 2.9. Ethics Statement

Embryos used in our experiments were not more than 6-days old. Hence, no license was required; the research complied with legal regulations (Directive 86/609/EEC and EU Directive 2010/63/EU). The above directives allow zebrafish embryos to be used in experiments up to the moment of free-living (approximately 5–7 dpf).

## 3. Results

### 3.1. Lethal Concentration (LC_50_) Determination

The toxic effect of THC is induced in a dose-dependent manner. The mortality rate rises as the concentration increases, as shown in Figure S1 of the Supplementary Material. The lethal concentration (LC_50_) was 1.54 mg/L while the LC_25_ and LC_75_ values were 1.27 mg/L and 2.09 mg/L, respectively.

### 3.2. Morphological and Function Alterations

**Figure 1:**
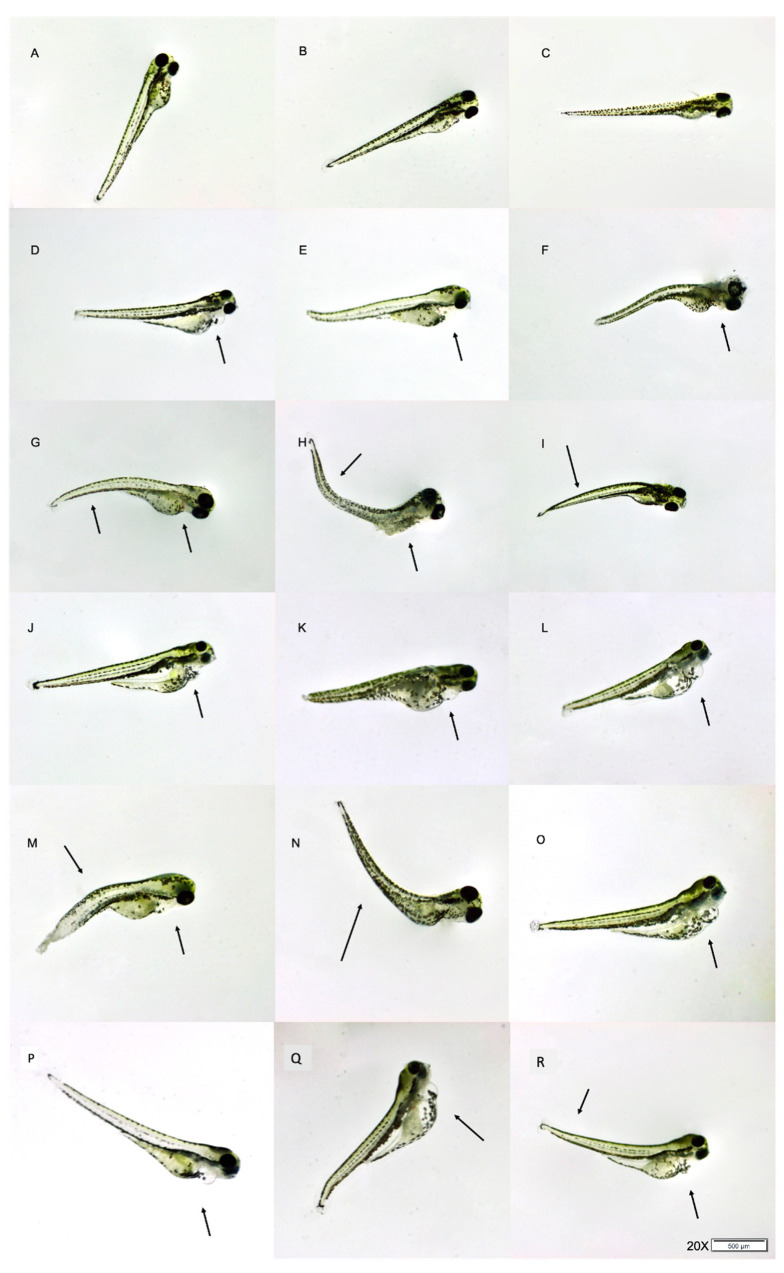
Morphological malformations of 5 dpf zebrafish exposed to THC. (**A**–**C**) Vehicle control larvae, (**D**–**F**) exposed to 1.00 mg/L, (**G**–**I**) larvae exposed 1.25 mg/L, (**J**–**L**) larvae exposed to 1.50 mg/L, (**M**–**O**) larvae exposed to 2.00 mg/L and (**P**–**R**) larvae exposed to 2.25 mg/L. Representative malformations are indicated by arrows.

The larvae were screened for malformations, following exposure to THC. The exposed zebrafish embryos showed dose-dependent morphological alterations while no morphological deformities were observed in the non-exposed larvae throughout the testing period (Figure 1A–C). The overall mortality of the non-exposed group was 3.1%. Embryos exposed to THC concentrations lower than LC_50_ presented insignificant developmental deformities, limited to minor tail bending and pericardial and yolk sac edemas (Figure 1). In contrast, above the LC_50_, the embryos exhibited obvious and progressively more pronounced phenotypic changes, such as bent and twisted notochord, and intense pericardial and yolk sac edema. 

The pericardial edema was observed at 24 hpf and became more apparent at 72 hpf. It is noteworthy that myocardial contractility was affected (decreased) after exposure to the highest concentration (Supplementary Material-Videos S1 and S2). Even at the lowest concentration of 1.00 mg/L, a marked decrease in blood flow was observed at 72 hpf. Specimens exposed to THC concentrations of 2.00 mg/L and 2.25 mg/L presented accumulation of blood cells in the blood vessels near the tail and degeneration of some body parts. The decay of the body was more obvious near the head, tail, and yolk sac. At the concentration of 2.25 mg/L, more than 80% of the surviving embryos showed severe body malformations. The yolk sac appeared to be deformed, the pericardial edema was prominent, and serious dose-dependent defects were noticed in the zebrafish heart. Also, the heart was lengthened, the chambers were distinguished without overlap, the atria appeared thinner and elongated and the ventricles were smaller than those of non-exposed larvae. These developmental disorders led most of the larvae to death after 5 dpf.

### 3.3. Heart Rate

**Figure 2:**
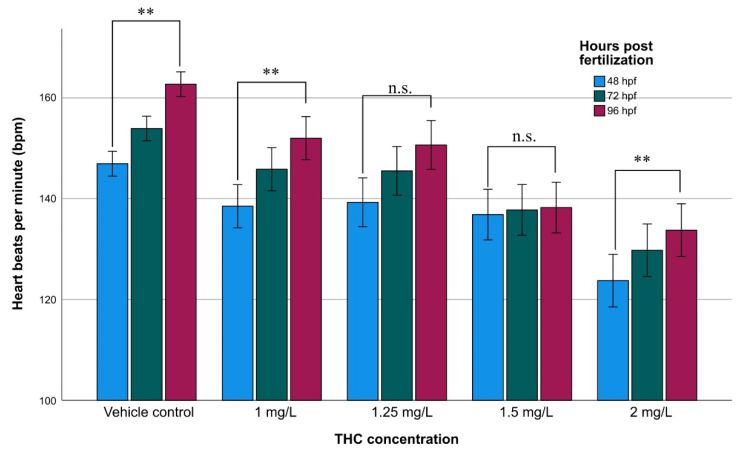
Heartbeat rate of zebrafish during embryonic development upon exposure to various concentrations of THC, at 48, 72, and 96 hpf. Asterisks denote statistically significant differences (**: *p* < 0.001, n.s.: non-significant) of heartbeat within various developmental stages at each concentration.

The mean heart rate values of the non-exposed group were 147 ± 3, 154 ± 3, and 163 ± 3 (Ν = 54) at 48, 72, and 96 hpf, respectively, showing a tendency for an increase during the ontogenetic development (Figure 2). The mean heart rate of the exposed embryos was significantly different from that of the non-exposed. The GLM analysis showed that heartbeat increased during embryonic development (*F* = 28.09; *p* < 0.001). It has been proved that THC influenced the heartbeat rate causing mild bradycardia. As the concentration increases the heartbeat decreases (*F* = 75.65; *p* < 0.001) while the combined effect (embryonic development × concentration of THC) was not statistically significant (*F* = 1.508; *p* = 0.153).

### 3.4. Behavioral Analysis

#### 3.4.1. Larval Activity

**Figure 3:**
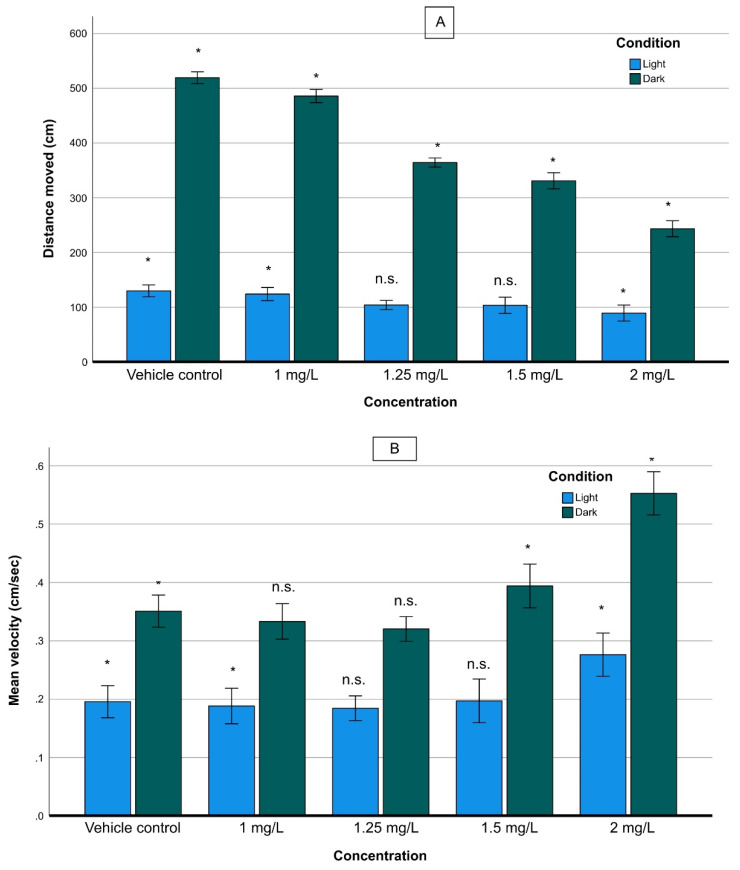
Distance moved (cm) (**A**), and velocity (cm/s) (**B**), of zebrafish larvae exposed to various THC concentrations under dark/light conditions. For the mean velocity (**B**) under dark conditions, non-statistically significant differences were observed between 1.0 and 1.25 mg/L of THC, while under light conditions non statistically significant differences were observed between 1.25 and 1.5 mg/L. (n.s.: non-statistically significant differences, *: statistically significant differences, *p* < 0.05).

Exposure to various THC concentrations demonstrated statistically significant differences in swimming velocity and total distance traveled between vehicle control larvae and those exposed to THC. The distance moved during dark was much longer than that in during light, for the same exposure time, at the same THC concentration. The exposure caused a significant reduction in larval locomotor activity in terms of distance moved (hypolocomotion), in dark/light cycling conditions (Figure 3A). Specifically, in both dark and light phases, the distance moved decreased significantly as the THC concentration increased. Also, the rate of reduction of the distance traveled as a function of the concentration of THC during the dark phase, (slope of the curve) was higher than that in the light phase. 

The GLM analysis supported that the concentration and the light/dark phases as well as the interaction of the two parameters, had a statistically significant effect on larval activity (concentration: *F*_(4.756)_ = 5703; *p* < 0.001, light-dark phases: *F*_(1.756)_ = 38,501; *p* < 0.001, interaction: *F*_(4.756)_ =1674; *p* < 0.001). The slope of reduction during the dark phase was significantly higher than that under light conditions, signifying that, in terms of distance moved, the behavior of zebrafish larvae under dark was affected more than in light (Figure 3A).

The velocity study (in cm/s) revealed more complex patterns than the behavior, already described above. In both dark and light conditions, there was a slight reduction in the velocity at relatively low concentrations of THC (<1.25 mg/L) and an increase at high ones (Figure 3B). As a general trend, the velocity in the dark phase was significantly higher than that in the light phase regardless of the THC concentration. As the concentration increased up to 1.25 mg/L the velocity decreased under both dark and light conditions and then, it increased when the concentration rose to 2.00 mg/L. A GLM analysis showed that concentration, light and the interaction of the two parameters had significant effect on larval activity (concentration: *F*_(4.756)_ = 77,148; *p* < 0.001, light/dark phases: *F*_(1.756)_ = 8422; *p* < 0.001, interaction: *F*_(4.756)_ =152; *p* < 0.001). 

With respect to the mobility of larvae in relation to the light/dark cycling, it was found that the larvae exposed to THC exhibited significant hypolocomotor activity in both phases, compared with the non-exposed group. The reduction of activity (in terms of distance moving) was proportional to the concentration of THC, as Figure 3 reveals. Interestingly, the difference in the distance moved between the two phases decreased as the concentration increased, indicating the inability of larvae to adapt themselves to the alternation between the two phases, under the influence of THC. 

**Figure 4:**
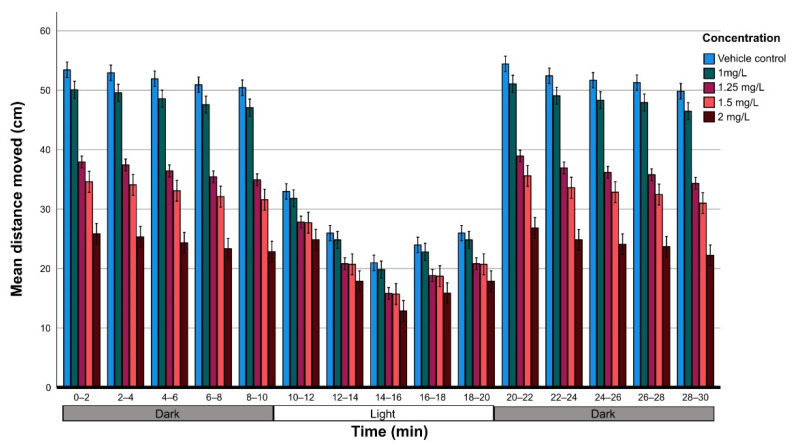
Motor response of zebrafish larvae at 6 dpf in relation to THC concentration; locomotor activity of 6-days-old post-fertilization vehicle control larvae (n = 79) and exposed to 1 mg/L (n = 67), 1.25 mg/L (n = 140), 1.5 mg/L (n = 44) and 2 mg/L (n = 45) were monitored in a 30-min period of dark/light cycling (0–10 min, dark; 10–20 min, light [white area]; 20–30 min, dark); the distance was measured at intervals of 2 min and was compared with the vehicle control.

Data on the average distance moved per concentration were analyzed using GLM analysis followed by the Bonferroni post hoc test (83 larvae for the non-exposed group and about 50 larvae per concentration) in relation to the light and dark conditions. The analysis showed that there were significant differences in the average distance because of the effect of the THC concentration (*F*_4,765_) = 325,22; *p* < 0.001) in relation to light conditions (*F*_1,765_ = 9881.25; *p* < 0.001). However, there was no statistically significant difference between the two dark phases (0–10 min and 20–30 min) suggesting that there was no habituation during the experimental period (Figure 4).

**Figure 5:**
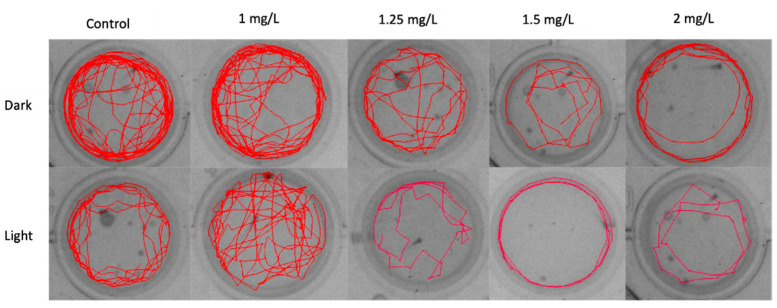
Track plot of zebrafish embryo movement in light and dark conditions, in relation to the concentration of THC.

The track plot of zebrafish larvae exposed to the THC showed a dose-dependent effect on the relative time spent in the inner zone (Figure 5). As the concentration increased larvae avoided moving in the inner zone of the wells, in both conditions, preferring to swim in the proximity of the walls.

#### 3.4.2. Touch Motor Response (TMR) and Vibrational Startle Response (VSR) 

**Figure 6:**
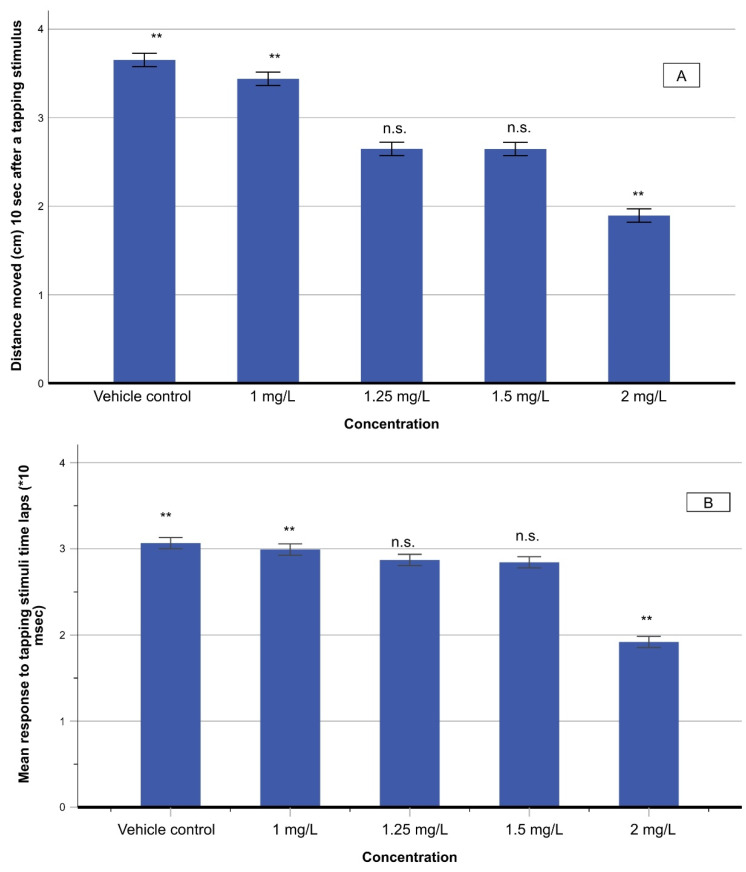
Distance moved (in cm) in 10 s (**A**) and response time (10^−3^ s) (**B**) after a stimulus delivery on zebrafish larvae exposed to various concentrations of THC. Asterisks denote statistically significant differences (**: *p* < 0.001, n.s.: non-significant).

The touch motor response (TMR) was conducted by provoking a light touch stimulus to the rostral head of zebrafish larvae by a glass capillary. Both non-exposed and THC-exposed larvae responded adequately, ensuring that their sensory systems were sufficiently developed. Subsequently, the VSR test was employed to check the difference in sensory development between non-exposed and THC-exposed larvae. The distance moved in 10 s and the response time after the stimulus delivery were measured to obtain an insight into the effect on the response and baseline swim, after tapping stimuli. A significant decrease in the swimming distance was noticed as the concentration of THC increased (*F*_(4,70)_ = 8748.12; *p* < 0.001) (Figure 6). The response time of zebrafish larvae in response to the applied stimulus decreased too, at elevated THC concentrations (*F*_(4,70)_ = 5212.8; *p* < 0.001) (Figure 6). 

### 3.5. Metabolomic Study

For the metabolomic study, the concentrations of 1.00, 1.125, and 1.25 mg/L were employed, as they induce low mortality, while, at the same time, multiple phenotypic and behavioral alterations appeared in the exposed zebrafish. 

Various metabolites were confirmed in each larvae sample hinting towards metabolic alterations after exposure, as can be seen in Table S1 of Supplementary Material. Also, spectroscopic data regarding the identification of the compounds are presented in Table S2 of Supplementary Material based on the NMR spectra of Figure S2. A total of twenty-two metabolites were identified. In the vehicle control, seventeen metabolites were identified, whereas in the larvae exposed to 1.00, 1.125, and 1.25 mg/L, fifteen, fifteen, and eight metabolites were identified, respectively. Certain metabolic pathways were perturbed following the exposure to THC and these alterations are responsible for the observed phenotype. In the vehicle control, fourteen active metabolic pathways were identified while in the larvae exposed to 1.00, 1.125, and 1.25 mg/L, fourteen, ten, and six pathways were identified, respectively. The complete list of the metabolic pathways is given in Table 1. 

## 4. Discussion

The field of human health has been invariably expanding to meet the needs of surveillance and prevention of consequences of exposure on both wildlife and human health. In this study, the effect of THC on zebrafish, used as a model organism, was evaluated following a multidimensional approach. The concentration-dependent toxicological, morphological, developmental, locomotor, and physiological effects were in agreement with the metabolomic pathways, after exposure for 96 h. Similar results have been shown concerning other common cannabinoids, such as cannabinol and CBD; however, the effects of THC seem to be more adverse [17,24,25]. The toxicity of THC (LC_50_ = 1.54 mg/L) was found to be lower than that found in other studies (3.37 mg/L and 3.65 mg/L) [16,24]. The dechorionation of eggs, adopted in our case, can account for this difference in toxicity data. In our study, the adverse effects include high mortality, morphological implications (pericardial and yolk sac edema, curved axis), reduction of cardiac contractility, and locomotor activity disorders of embryos. Embryos of the non-exposed group did not present any malformation. By contrast, THC-exposed embryos showed dose-dependent morphological alterations. Embryos exposed to THC concentrations lower than LC_50_ (<1.54 mg/L) showed mild deformities. This was not the case for higher concentrations, where severe deformities were provoked. Pericardial edema was observed at 24 hpf and this was more severe at 72 hpf. 

Zebrafish embryos are suitable to explore research questions about the effect of drugs (e.g., THC), pollutants, and other substances on heart development and function, mainly due to the external development of the transparent embryos, the well-characterized developmental stages of the heart, and the easiness of treatment of embryos during the heart development. Exposure to various concentrations of THC produced progressive dose-dependent effects. 

Heart rate increased during the early development (through 48 hpf to 96 hpf) regardless of the THC concentration and decreased with the increase in the concentration (Figure 2), manifesting progressive mild bradycardia. We have already demonstrated that cannabinol, the main metabolite of THC, causes cardiac defects [17]. The observed bradycardia between exposed and non-exposed larvae could be explained by the abnormal heart development (Figure 1) and function, as described above.

The heart development and its function were significantly affected by the exposure to THC. Even at the lowest concentration of 1.00 mg/L, pericardium edema was observed, which was more obvious as the concentration increased over time (Figure 2). Heart failure and morphological alterations provoked improper heart chambers development (Figure 1). The chambers were elongated and the heart appeared to be string-like and shorter in width. Moreover, the ventricle and the atrium were separated, which is contrary to the overlapped champers of non-exposed embryos. These alterations, according to Chen et al. [26], could be attributed to deregulated cardiovascular development genes (krit1). The developmental disorders led to the death of most of the larvae, past 5 dpf. From the metabolomic study, it appeared that perturbation in pyrimidine metabolism and aminoacyl-tRNA biosynthesis occurred (Table 1). The imbalance of the pyrimidine metabolism can affect brain development and cause growth retardation and epilepsy [27]. Downregulation of the aminoacyl-tRNA biosynthesis metabolic pathway leads to insufficient tRNA synthesis, which leads to decreased production of proteins [28]. 

Severe malformation and dysfunctions related to the development and function of the heart as well as to the circulatory system seem to be correlated with the metabolic pathways of pentose and glucuronate interconversions (Table 1). It was found that these metabolic pathways were affected at high concentrations of THC. The highest mortality of zebrafish at the concentration of 1.25 mg/L can be ascribed to cardiotoxicity induced by perturbations of this pathway [29]. The progressively increasing appearance of pericardial edema and the accumulation of blood cells in the blood vessels near the head, tail, and yolk sac at higher concentrations (2.00 and 2.25 mg/L) are attributed to the downregulation of the pyrimidine metabolic pathway, which is known to be involved in inflammatory processes. It is well documented that disorders of pyrimidine metabolism are the cause of a very large number of disorders, seizures, and mental retardation among others [27,30,31]. Moreover, the metabolomic analysis (Table 1) supports that the affected metabolomic pathway of pentose and glucuronate, which takes place after exposure to higher concentrations, affects the development of the ventricular system and heart further to the supply of myocardial oxygen [29]. It is most likely that certain of the above factors induced alterations, culminating in the impairment of cardiac contractility (Figure 2), at the highest exposure concentration of THC (Supplementary-Video S2).

### 4.1. Behavior

An alteration of the behavioral profile of the studied larvae was monitored, as a result of the combination of depletion of energy reserves, central nervous system disorders, and anxiogenic and anxiolytic responses. Typically, zebrafish larvae show hyperactivity during the dark phases and relative hypoactivity during the light phase, as exemplified by the distance moved and velocity. This behavior is altered under the effect of THC. It was proved that the distance moved decreased as the THC concentration rose, in an inversely proportional relationship (Figure 3A). The rate of reduction was higher in the dark than in the full light phase. The reduced distance covered by zebrafish indicates hypoactivity at elevated THC concentrations, which could be attributed to the morphological malformations affecting swimming ability. However, it could, also, be indicative of a sedative effect. According to Hartsel et al. [32], Δ9-THC suppresses locomotor activity and induces catalepsy (immobility) in healthy mice or rats, associated with CB1-related effects. The observed decrease in larval activity under both dark and light conditions could be attributed to the anxiolytic effect of THC [33], as reported for other animal models [34,35,36].

In regard to the velocity of zebrafish, a significant reduction was observed after exposure to concentrations up to 1.25 mg/L; this is not the case when the concentration increases (Figure 3B). During the dark phase, zebrafish larvae presented velocities significantly higher than those in the light phase. Overall, at higher concentrations (>1.25 mg/L), zebrafish larvae velocities were significantly higher than those in the non-exposed group although the distances moved were shorter. The increase in velocity may indicate hyperactivity. It has been proven that hyperactivity and hypoactivity associated with minor changes in the treatment procedure are indicative of anxiety-like behaviors in adult fish and rodents [37]. Similar, substantial effects have been observed earlier for zebrafish treated with cannabinol [17]. Finally, our findings on the total distance covered and velocity have proven that zebrafish embryos lack swimming activity and ability as the THC concentration rises. Interestingly, they become more stressed by reaching the highest velocity and moving for shorter periods, at the highest concentration.

The track plot of zebrafish larvae (Figure 5) shows progressive anxiogenic effects of THC in alternate dark/light conditions and larvae avoid moving to the center of the well as the concentration of THC increases. Swimming in close proximity to the tank walls is an established behavioral attribute of anxiety in fish and is increased in a high-anxiety state. The fact that zebrafish remains longer near the walls irrespective of the concentration of THC, confirms the perception that it is a photophobic organism [38]. However, this behavior becomes more obvious under the influence of THC and is reflected in the mobility, expressed by the distance moved in dark and light (Figure 4). The effect of cannabinoids on stress and anxiety is not clear due to the activation of the endocannabinoid system, which may lead to anxiogenic and anxiolytic responses. These disorders are associated with several complex interactions among cannabinoids, receptors, and distinct neural pathways [39]. 

The metabolomic analysis, additionally, shows alterations in pantothenate and CoA biosynthesis (Table 1), where CoA is synthesized as an important cofactor of many cellular processes. This pathway is upregulated when zebrafish are exposed to 1.00 and 1.125 mg/L THC but this is not the case in the vehicle control and the sample treated with 1.25 mg/L of THC. Downregulation of this pathway is related to neurodegeneration with symptoms including spasticity [40]. In our case, this pathway is probably upregulated in order for larvae to cope with neurodegeneration. This notion is strengthened by the different velocities observed in relevant experiments. Spasticity results in hyperreflexia, which is the state of over-responsive reflexes. When zebrafish are exposed to 1.00 and 1.25 mg/L of THC a decreased velocity is observed compared with vehicle control fish, which may be due to the upregulation of pantothenate and CoA biosynthesis pathway. When zebrafish are exposed to 1.25 mg/L of THC their velocity increases compared with vehicle control fish, presumably due to hyperreflexia. Behavioral responses, such as lower distance movement and changes in velocity (diminished at low concentrations and sharply increased at higher concentrations) may also be linked to either of the following: (i) lack of energy due to downregulation of aminoacyl-tRNA biosynthesis, which leads to insufficient t-RNA biosynthesis and decreased production of proteins, (ii) fatty acid degradation (Table 1), (iii) combination of the above effects (stress, anxiety, downregulation energy metabolic pathways). Moreover, at all tested concentrations of THC, the biotin metabolism pathway of larvae is downregulated. Biotin is an important cofactor for many metabolic pathways, such as fatty acid metabolism and the regulation of gene expression in eukaryotic cells [41,42]. Deficiency of biotin can result in mental retardation, developmental delay, and the development of Leigh.

The reduction of response time, after an external stimulus with the tapping device, is indicative of increased sensitivity (Figure 6), which can be ascribed to stress and anxiety induced by the rise of THC concentration. This agrees with the progressive increase in the velocity (Figure 3B). These results indicate fast-start swimming activity that becomes faster as THC concentration increases. This behavior, known as fast-start readiness, is a common effect when testing drugs using zebrafish as a model organism. It has been proved that these responses are linked to anxiety and fear behavior of zebrafish [33]. To further support the above finding, an analysis was carried out by applying a startle to motor function, sensory physiology, and basic forms of learning of zebrafish [43]. The zebrafish larvae tend to follow a pattern of rapid contractions of axial musculature. A behavioral approach to the startle response measurements is that THC exposure induces hyperactivity in response to a sensory startle. This manifests itself in the reduction of reaction time (Figure 6B). Metabolomic analysis shows that alterations of D-glutamine and D-glutamate metabolic pathways hint towards disequilibrium in neurotransmitter systems, which can lead to neurotoxicity and/or neuronal dysfunction [17].

### 4.2. Metabolic Alterations

As can be seen in Table 1, many metabolic pathways have been perturbed after exposure to THC. Even at the lowest THC concentration of 1.00 mg/L, phenotypic alterations were evident and associated with increased mortality at higher concentrations. These alterations are responsible for the observed phenotype. Metabolomic analysis showed even more effects on metabolic pathways than those discussed above in the manuscript. Downregulation of the steroid biosynthesis pathway was observed in all cases of the tested concentrations of THC. This pathway is of utmost importance for the maturation of fish gonads and sexual maturation. It is expected that issues may arise in the sexual maturation of fish after exposure, which may result in unsuccessful reproduction during the reproductive season [44].

The fatty acid degradation pathway was activated to produce energy from fatty acids. This pathway has also been proven to be activated in cancer, as an increased cell proliferation results in a higher demand for fatty acids for the synthesis of cellular components [45].

Glutathione metabolism produces glutathione, a very efficient antioxidant compound that can scavenge and reduce reactive oxygen species. The activation of this pathway provides tangible proof that the THC induces oxidative damage to cells that need to be protected. This is strengthened further by the fact that methionine was detected in the samples exposed to the two lower concentrations. Methionine is not only important for the synthesis of glutathione but also protects DNA from oxidative damage [46].

The abovementioned alterations along with those reported in the section of Results bespeak a deleterious effect of THC on the metabolism of zebrafish, which is ultimately reflected in the phenotype and the behavior of the zebrafish. It is of particular importance to know beforehand these consequences in order not only to prevent unwanted side-effects from THC usage but also to take precautionary measures for a potentially hazardous substance to the environment.

## 5. Conclusions

As the pharmacological properties and therapeutic applications of *Cannabis sativa* L. pace with the upsurge of interest of the scientific community in harnessing its constituent phytocannabinoids, the illicit use may raise serious health issues. In this study, we explored the effect of THC on zebrafish embryos in an acute exposure regime by studying the systemic toxicity, behavioral activities and metabolic pathways, and fundamental functions during critical developmental stages. The preliminary results showed that THC can cause multiple adverse health effects on the organism after exposure at various concentrations. Changes were observed in the behavior of zebrafish including alterations in their locomotor activity and sensitivity to light/dark changes. In addition, the metabolomic study, not only provided substantiating evidence of certain experimental findings and explained the alterations at a metabolomic level but also hinted towards underlying human health effects that may arise in the near future. Due to the growing demand for cannabis-based products, the results highlight the need to consider the long-term ramifications of early-life exposure to cannabinoids. As jurisdictions increasingly permit the use of Cannabis for medical and other purposes, this research provides critical insight into the consequences of the use of phytocannabinoids. In this context, more research will be needed to identify the exact mechanisms for the detrimental effects versus the beneficial role of THC.

## Figures and Tables

**Table 1:** Metabolic pathways identified in the vehicle control sample and the larvae exposed to various THC concentrations.

Metabolic Pathway	Vehicle Control	THC 1.00 mg/L	THC 1.125 mg/L	THC 1.25 mg/L
Amino sugar and nucleotide sugar metabolism	✓	✓	✓	✓
Cysteine and methionine metabolism	✓	✓	✓	✓
Riboflavin metabolism	✓	✓	✓	✓
Starch and sucrose metabolism	✓	✓	✓	✓
Galactose metabolism	✓	✓	✓	✓
Neomycin, kanamycin, and gentamicin biosynthesis	✓	✓	✓	✓
Pentose and glucuronate interconversions	✓	✓	✓	
Pyrimidine metabolism	✓	✓	✓	
Aminoacyl-tRNA biosynthesis	✓	✓		
Arginine and proline metabolism	✓	✓		
D-Glutamine and D-glutamate metabolism	✓	✓		
Nitrogen metabolism	✓			
Steroid biosynthesis	✓			
Biotin metabolism	✓			
Pantothenate and CoA biosynthesis		✓	✓	
Fatty acid degradation		✓	✓	
Glutathione metabolism		✓		

## Data Availability

Data concerning anything from the current project can be given from the corresponding author: Constantine D. Stalikas, cstalika@uoi.gr.

## Supplementary Materials

The following supporting information can be downloaded at: https://www.mdpi.com/article/10.3390/molecules27175506/s1, Experimental S1: Zebrafish toxicity testing; Vibrational startle response (VSR); Metabolomic study and data processing-Instrumentation [47]; Figure S1: Mortality pattern of zebrafish larvae exposed to THC; Figure S2. NMR spectra of the metabolomes of vehicle control larvae (blue spectrum) and larvae exposed to 1.00 mg/L (red spectrum), 1.125 mg/L (green spectrum), and 1.25 mg/L (purple spectrum) of THC. Table S1: Metabolites detected in vehicle control larvae and larvae treated with THC; Table S2: Spectroscopic data employed for the identification of the metabolites in larvae; Video S1: Cardiac function of a non-exposed larva; Video S2: Cardiac function of a larva exposed to 2 mg/L of THC.

## Sample Availability

Not available.
