# Supplementary material for: In the Swim of Cannabis: Developmental Toxicity and Metabolomic Pathway Alterations of Zebrafish Larvae Exposed to THC for the Assessment of Its Potential Environmental and Human Health Impact
Source: Molecules. 2022 Aug 27;27(17):5506. doi: 10.3390/molecules27175506 (PMC9458094; doi:10.3390/molecules27175506)
Supplement: Supplementary file 1 [file molecules-27-05506-s001.zip › Supplementary material THC- 3-8-2022.pdf]

In the swim of cannabis: developmental toxicity and metabolomic pathway alterations of zebrafish larvae exposed to THC for the assessment of its potential environmental and human health impact

Theodoros Chatzimitakos[a], Ieremias Chousidis[b], Dimitrios Leonardos[c], Constantine Stalikas[a*], Ioannis Leonardos[b]

[a] Laboratory of Analytical Chemistry, Department of Chemistry, University of Ioannina, Ioannina 45110, Greece

[b] Laboratory of Zoology, Biological Applications and Technology Department, University of Ioannina 45110, Greece

[c] School of Medicine, University of Ioannina, Ioannina 45110, Greece

Experimental

Zebrafish toxicity testing

The dechorionated embryos were placed in 24-well culture plates (2 embryos per well, 1.5 mL of solution per well) and each experiment was performed in triplicate. Preliminary tests were performed to evaluate the range of 0%-100% mortality. The concentration range was found to vary from 1.00 mg/L to 2.50 mg/L and E3 medium embryo buffer was used as vehicle vehicle control (non-exposed). In the current study, five different concentrations of THC were tested (1.00, 1.25, 1.50, 2.00, 2.25 mg/L). In total, 1157 embryos were studied, of which 384 belonged to the non-exposed group. Twelve embryos of the non-exposed group were found to be dead (3.1%) due to the manipulations during the dechorionation. This percentage was further used to assess toxicity.

Each experiment lasted about 96 post-fertilization time (hpf) and began at 24 hpf, approximately at 26-somite point, according to Kimmel et al. [1]. During the experiments, toxicity, morphological abnormalities including yolk-sac edema, spinal curvature, tail deformity,

uninflated swim bladder, and cardiac defects were recorded every 24 h, using a stereoscope (Olympus BX43) equipped with a digital camera, and high-resolution pictures were taken to be processed with the Image-Pro Plus software (Image-Pro Plus 7, Media Cybernetics). The recordings were performed at the same time (10 am) every day and lasted until the end of the experiment.

Vibrational startle response (VSR)

The video tracking and the escape response were analyzed using EthoVision XT14 software (Noldus, Wageningen, The Netherlands). Embryos were transferred from the colony room to the testing room on the day of the test, to acclimate before each recording. The DanioVision Temperature Control Unit (DVTCU-0011) kept the temperature constant at 28°C. Trials were performed in 24 well plates, with one larva in each well containing 1,5 mL of E3 embryo medium. After 1 h of acclimatization, videos of 2 min were acquired at 60 frames per second, in full dark. The video recordings and the escape response were analyzed for each larva by measuring the distance covered in 10 sec and the response time to the tapping after triggering the stimulus. For statistical analysis, the built-in software Noldus Inc., EthoVision was used. The differences were considered significant at $p<0.001$ and marginally significant at $p<0.05$.

Metabolomic study and data processing

Instrumentation

^{1}H-NMR experiments were carried out using a Bruker AV-500 spectrometer equipped with a TXI cryoprobe (Bruker BioSpin, Rheinstetten, Germany). System control and spectra processing were carried out using the TopSpin 2.1 software. Spectra were recorded at 298 K. Chemical shifts were reported using TSP-d4 (0.02 mmol/L) as reference. Acquisition time was set at 4.1 sec and relaxation delay 5 sec, using 90° pulse length. In addition, 64 K points were used and 256 scans were recorded. After Fourier transform, phase and baseline were manually corrected. For the chromatographic separation of the metabolites, a UHPLC Accela LC system (Thermo Fisher Scientific, Inc. GmbH, Bremen, Germany) was used. The chromatographic column was a Hypersil GOLD 1.9 µm particle size (100 mm × 2.1 mm I.D), kept at 30°C. Water (A) and acetonitrile (B),

both acidified with 0.1% (v/v) formic acid were used for the gradient elution. The flow rate was set at 300 μL min^{-1}. The elution program was as follows: 0-13.78 min, 20-90% B, 13.78-15.28 min, 90% B, 15.28-18.06 min, 90-20% B, followed by a 2-min re-equilibration time of the column. A hybrid linear trap quadrupole (LTQ) Orbitrap mass spectrometer (LTQ-Orbitrap XL 2.5.5 SP1, Thermo Fisher Scientific, Inc. GmbH, Bremen, Germany), equipped with an Electrospray Ionization (ESI) source, was used for metabolite detection. Nitrogen was used as the sheath and auxiliary gas to deliver effluents to the ion source. Both ionization modes were employed. When positive ionization mode was employed, 2.5 μL of the sample was injected, and source voltage, tube lens, heated capillary voltage, and temperature were set at 3.40 kV, 110 V, 40.00 V, and 320 °C, respectively. In the negative ionization mode, 10 μL of the sample was injected and source voltage, tube lens, heated capillary voltage, and temperature were set at 3.70 kV, 120 V, -30.00 V, and 320 °C, respectively. In both cases, a full scan mode (60000 resolution and m/z range: 50-1000) and a most-intense-ion scan mode (MS/MS fragmentation of the most abundant ion) (7500 resolution) were used. Instrument operation, data recording, and processing were carried out using the Thermo Xcalibur 2.1 software.

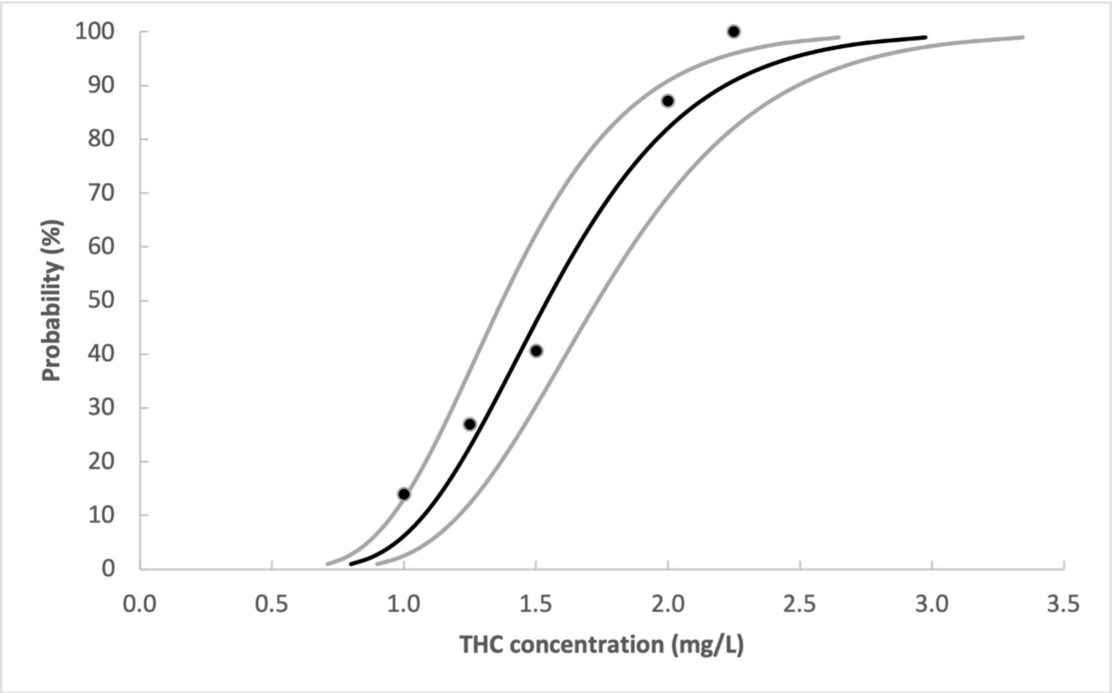

Figure S1. Mortality pattern of zebrafish larvae exposed to different concentrations of THC up to 96 hpf. The LC_{50} value was 1.54 mg/l, while LD_{25} was 1.27 mg/l and LD_{75} was 2.09 mg/l.

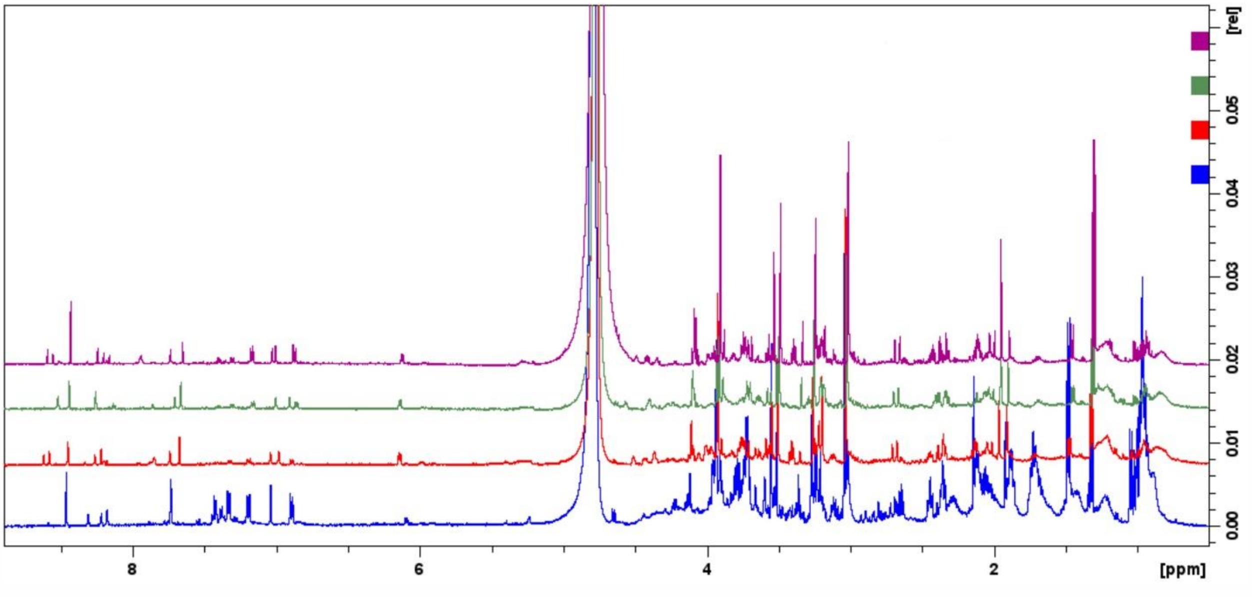

Figure S2. NMR spectra of the metabolomes of vehicle control larvae (blue spectrum) and larvae exposed to 1.00 mg/L (red spectrum), 1.125 mg/L (green spectrum) and 1.25 mg/L (purple spectrum) of THC.

Table S1: Metabolites detected in vehicle control larvae and larvae treated with THC.

**Metabolite**	**Vehicle control**	**THC 1.00**	**THC 1.125**	**THC 1.25**
4-Hydroxyproline	✓	✓		
Alpha-Lactose	✓	✓	✓	✓
Biotin	✓		✓	
Cellobiose	✓	✓	✓	✓
Cholecalciferol	✓			
Coenzyme A		✓	✓	
D-Galactose	✓	✓	✓	✓
D-Glucose	✓	✓	✓	✓
D-Mannose	✓			
D-Xylose	✓	✓		
Flavin mononucleotide	✓	✓	✓	✓
L-Glutamine	✓	✓		
L-Homoserine	✓	✓		
L-Lysine	✓			

L-Methionine		✓	✓	
NADPH			✓	
Nicotine	✓	✓	✓	
S-Adenosylhomocysteine	✓	✓	✓	✓
Stachyose	✓	✓	✓	✓
Thymidine	✓	✓	✓	
Uridine diphosphate glucuronic acid			✓	
Uridine diphosphate-N-acetylglucosamine			✓	✓

Table S2: Spectroscopic data employed for the identification of the metabolites in larvae.

metabolite	theoretical mass	experimental mass	difference (ppm)	MS/MS fragmentation	^{1}H NMR characteristic signals
4-Hydroxyproline	131.1299	131.1293	-4.57	114.0549/96.0443	1.77 (ddd, J = 14.4, 7.0, 4.3 Hz), 2.16 (ddd, J = 14.4, 8.1, 7.4 Hz), 3.22 (dd, J = 9.3, 8.3 Hz)
Alpha-Lactose	342.2965	342.2972	2.04	145.0495/103.0389/91.0389	3.17 (t, J = 10.2 Hz), 3.54 (dd, J = 10.2, 3.5 Hz)
Biotin	245.3183	245.3184	0.41	210.058/199.0899/157.0430	2.20 (t, J=7.43 Hz)
Cellobiose	342.2965	342.2954	-3.21	281.086/181.0706/163.0600/	3.49 (dt, J = 10.3, 6.4 Hz), 3.81 (d, J = 6.4 Hz)
Cholecalciferol	384.6377	384.6370	-1.82	367.3365/261.2582	1.54 (dddd, J = 13.0, 3.0, 2.5, 2.2 Hz)), 2.42 (dd, J = 14.1, 10.3 Hz)

Coenzyme A	768.5413	768.5415	0.26	708.1191/654.1506/564.0656 /261.1267/136.0617	0.73 (s), 0.85 (s), 3.99 (s), 8.25 (s)
D-Galactose	181.1632	181.163	-1.1	145.0495/103.0389/91.0389	3.92(d, J=3.07 Hz)
D-Glucose	180.1559	180.1555	-2.22	163.0600/145.0495/103.0389 /91.0389	4.79 (d, J = 2.6 Hz), 3.79 (d, J = 6.4 Hz)
D-Mannose	180.1559	180.1563	2.22	145.0495/103.0389/91.0389	9.61 (d, J = 6.9 Hz), 4.58 (dd, J = 6.9, 4.5 Hz)
D-Xylose	150.1299	150.1293	-3.99	115.0389/91.0389	3.64 (dd, J = 14.2, 10.3 Hz), 4.42 (d, J = 10.3 Hz)
Flavin mononucleotide	457.3511	457.351	-0.22	255.0876/237.0770/212.0818	7.63 (d, J=2.86 Hz), 7.68 (s)
L-Glutamine	146.1445	146.1448	2.05	101.0709/84.0443	2.32 (t, J = 7.4 Hz)
L-Homoserine	119.1192	119.1189	-2.51	102.0549/84.0443	2.05 (td, J = 7.2, 2.7 Hz)
L-Lysine	147.1949	147.1947	-1.36	130.0862/101.1073/84.0807	3.74 (t, J=6.09 Hz)
L-Methionine	149.2110	149.2108	-1.34	104.0528/88.0215	2.19 (s), 2.64 (t, J = 2.7 Hz)

NADPH	745.4209	745.4220	1.47	397.0202, 134.0467	4.17 (dt, J = 4.4, 3.3 Hz)), 6.33 (d, J = 7.7 Hz)
Nicotine	162.2316	162.2323	4.31	145.0766/78.0344	2.39 (s), 3.88 (dd, J = 9.3, 5.4 Hz)
S-Adenosylhomocysteine	384.4110	384.4114	1.04	220.0638/136.0617/119.0352	6.30 (d, J = 7.7 Hz), 7.87 (s), 8.50 (s)
Stachyose	666.5777	666.5777	-0.75	649.2185/487.1657/325.1129 /181.0706/163.0600	4.12 (d, J = 6.3 Hz), 4.45 (d, J = 2.7 Hz)
Thymidine	243.2359	243.2362	1.23	169.0607/127.0502/109.0396 /97.0396/82.0287	1.88 (s), 3.97 (s)
Uridine diphosphate glucuronic acid	580.2853	580.2859	1.03	377.0145/227.0.662/113.0345	6.21 (d, J = 9.3 Hz), 7.61 (d, J = 10.8 Hz)
Uridine diphosphate-N-acetylglucosamine	607.3537	607.3537	0.49	539.0673/364.0193/284.0529 /113.0345/83.0239	5.94 (d, J = 2.6 Hz), 7.62 (1H, d, J = 10.8 Hz)

Video S1: Cardiac function of a non-exposed larva

Video S2: Cardiac function of a larva exposed to 2 mg/l of THC

References

1. Kimmel, C.B.; Ballard, W.W.; Kimmel, S.R.; Ullmann, B.; Schilling, T.F. Stages of Embryonic Development of the Zebrafish. *Dev Dyn* **1995**, *203*, 253–310, doi:10.1002/aja.1002030302.
